# Characterization and Functional Properties of Gastric Tissue-Resident Memory T Cells from Children, Adults, and the Elderly

**DOI:** 10.3389/fimmu.2014.00294

**Published:** 2014-06-19

**Authors:** Jayaum S. Booth, Franklin R. Toapanta, Rosangela Salerno-Goncalves, Seema Patil, Howard A. Kader, Anca M. Safta, Steven J. Czinn, Bruce D. Greenwald, Marcelo B. Sztein

**Affiliations:** ^1^Center for Vaccine Development, University of Maryland School of Medicine, Baltimore, MD, USA; ^2^Department of Pediatrics, University of Maryland School of Medicine, Baltimore, MD, USA; ^3^Department of Medicine, University of Maryland School of Medicine, Baltimore, MD, USA; ^4^Division of Gastroenterology and Hepatology, University of Maryland School of Medicine, Baltimore, MD, USA

**Keywords:** LPMC, stomach, gastric tissue-resident/memory T cells, multifunctionality

## Abstract

T cells are the main orchestrators of protective immunity in the stomach; however, limited information on the presence and function of the gastric T subsets is available mainly due to the difficulty in recovering high numbers of viable cells from human gastric biopsies. To overcome this shortcoming we optimized a cell isolation method that yielded high numbers of viable lamina propria mononuclear cells (LPMC) from gastric biopsies. Classic memory T subsets were identified in gastric LPMC and compared to peripheral blood mononuclear cells (PBMC) obtained from children, adults, and the elderly using an optimized 14 color flow cytometry panel. A dominant effector memory T (T_EM_) phenotype was observed in gastric LPMC CD4^+^ and CD8^+^ T cells in all age groups. We then evaluated whether these cells represented a population of gastric tissue-resident memory T (T_RM_) cells by assessing expression of CD103 and CD69. The vast majority of gastric LPMC CD8^+^ T cells either co-expressed CD103/CD69 (>70%) or expressed CD103 alone (~20%). Gastric LPMC CD4^+^ T cells also either co-expressed CD103/CD69 (>35%) or expressed at least one of these markers. Thus, gastric LPMC CD8^+^ and CD4^+^ T cells had the characteristics of T_RM_ cells. Gastric CD8^+^ and CD4^+^ T_RM_ cells produced multiple cytokines (IFN-γ, IL-2, TNF-α, IL-17A, MIP-1β) and up-regulated CD107a upon stimulation. However, marked differences were observed in their cytokine and multi-cytokine profiles when compared to their PBMC T_EM_ counterparts. Furthermore, gastric CD8^+^ T_RM_ and CD4^+^ T_RM_ cells demonstrated differences in the frequency, susceptibility to activation, and cytokine/multi-cytokine production profiles among the age groups. Most notably, children’s gastric T_RM_ cells responded differently to stimuli than gastric T_RM_ cells from adults or the elderly. In conclusion, we demonstrate the presence of gastric T_RM_, which exhibit diverse functional characteristics in children, adults, and the elderly.

## Introduction

In human, peripheral blood memory T (T_M_) cells are commonly grouped into two major subsets based on their functional status and expression of defined homing receptors (e.g., CD62L, CCR7, and CD45RA) (1): central memory T (T_CM_) and effector memory T (T_EM_) cells. While T_CM_ cells express the lymph node-targeting molecules CD62L and CCR7, T_EM_ cells largely lack these receptors, and typically express defined homing molecules that endows them with the ability to migrate to peripheral non-lymphoid tissues (1, 2). Recently, a novel population of T cells known as tissue-resident memory CD8^+^ T (T_RM_) cells has been described. These T_RM_ cells have the ability to remain for long periods of time in peripheral tissues (e.g., intestinal and vaginal mucosa, skin, brain, and salivary glands) after pathogenic clearance. These cells have been shown to be antigen-specific, express markers of CD8^+^ T_EM_ cells, and their survival appears to be antigen-independent (3–6). Studies in epithelial and neuronal tissues have shown that T_RM_ are characterized by their expression of high levels of CD103 (the α-chain of the integrin αEβ7) and CD69 (a surface molecule typically found on recently activated T cells) (7, 8). While initially described in mice, these cells have been recently also identified in human tissues (9).

The stomach’s primary function is to digest food. With its low pH environment, the stomach has a secondary function in limiting the number of microorganisms that enter the intestinal tract. However, some microorganisms such as *Helicobacter pylori* (*H. pylori*) can cause significant pathogenesis and have a niche in this harsh environment. Various immune cells have been identified in stomach biopsies obtained during esophagogastroduodenoscopy (EGD) procedures. Immune populations described in gastric lamina propria mononuclear cells (LPMC) include γδT cells (10), CD13^+^ macrophages (Mϕ) (M1 and M2) (11), dendritic cells (DC) (12), natural killer (NK) (13), NK-T (14), neutrophils, B cells (15), and T (CD4^+^ and CD8^+^) cells (14, 16, 17). Various studies have demonstrated that intestinal immune cells (innate and adaptive) are phenotypically and functionally different from their systemic counterparts. For example, intestinal Mϕ is more phagocytic and bactericidal but secrete less pro-inflammatory cytokines than their peripheral blood counterparts (18). Additionally, the CD4^+^/CD8^+^ ratio is inverted (~1:3) in gastric LPMC (from healthy volunteers) compared to peripheral blood mononuclear cells (PBMC) (~3:1) (16, 19, 20). Despite these observations, very little is known about the different T cell subsets present in the gastric mucosa of healthy humans. For example, it is currently unknown whether T_CM_ and T_EM_ cells have similar percentage distribution in the gastric mucosa as in peripheral blood. Furthermore, despite that the acidic environment of the stomach provides a different environment than the one found in the mucosa of the small and large intestines, the gastric mucosa is part of the digestive tract and therefore has the potential to harbor T_RM_ cells. However, the presence of T_RM_ cells and their ability to exhibit effector functions (e.g., cytokine production and cytotoxicity) is currently unknown. In addition, no study has assessed the frequency of these cells in the gastric mucosa and differences in various age groups (children, adults, and the elderly). In the present study, after optimizing an isolation method for LPMC from gastric biopsies, we characterized in depth the memory CD4^+^ and CD8^+^ T cell subsets in gastric LPMC of healthy human volunteers. We demonstrated that the most abundant CD8^+^ T cell population in the stomach (>80%) was T_RM_ (CD62L^−^, CD45RA^−^, CD103^+^, and CD69^+^). These cells were able to produce various cytokines (either single or multiple cytokines simultaneously) when stimulated with mitogens and demonstrated differences in the strength and quality of the responses among different age groups (adults, children, and the elderly). These findings were only partially mirrored by gastric CD4^+^ T_RM_ cells, since only ~35% of the cells showed co-expression of CD103 and CD69. However, these cells were also able to produce cytokines and showed differences among the age groups evaluated. These novel findings suggest that T_RM_ might play a key role in protection from gastric infections and offer new insights into age differences in gastric immunity.

## Materials and Methods

### Volunteers and isolation of peripheral blood mononuclear cells

Volunteers were recruited from the Baltimore–Washington area and University of Maryland, Baltimore campus. Written informed consent was obtained and all procedures were approved by the University of Maryland, Baltimore IRB. Immediately after blood draws, PBMC were isolated by density gradient centrifugation and used freshly for stimulation and characterization. Blood and gastric biopsies were collected at the same time. A total of 57 volunteers (aged 7–85 years) were evaluated.

### Isolation of lamina propria mononuclear cells

**Figure 1:**
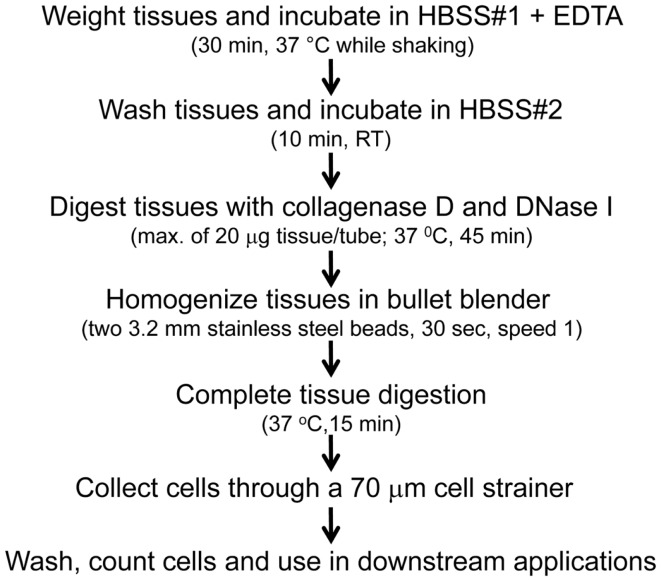
**Methodological diagram for the isolation of gastric LPMC**. A detailed description of the optimized procedure for isolation of LPMC is found in the Section “Materials and Methods.”

Gastric biopsies were collected from volunteers (7–85 years-old) referred for outpatient diagnostic upper endoscopy (EGD) at the University of Maryland Medical Center. The indications for EGD included abdominal pain, heartburn, GERD, dysphagia, and acute gastritis. Diagnostic pathology reports showed that the stomach’s antrum mucosa was either normal (*n* = 12) or exhibited mild inflammation (*n* = 45). No concurrent GI disease/disorders or other illnesses that may affect the GI tract were present. Additionally, all volunteers were confirmed to be *H. pylori* negative as determined by culture and rapid Urease test (CLO test).Tissue samples collected during EGD were transported to the laboratory facilities in a tube containing RPMI 1640 (Gibco, Carlsbad, CA, USA) with antibiotics/antifungal (Penicillin/Streptomycin/Amphotericin B; Gibco) and processed immediately after collection as shown in Figure 1 and Figure S1 in Supplementary Material. We first compared two methods the isolation of gastric LPMC: (i) a conventional method (CM) and (ii) bullet blender (BB) method. The CM method consisted of three steps: (a) removal of intraepithelial lymphocytes (IEL) [HBSS + EDTA (1 mM)], (b) digestion of the resulting tissues (collagenase D/DNase I), and (c) disaggregation of the tissues (by teasing of the tissues between the frosty ends of two microscope glass slides). The BB method also consisted of three steps. The first two steps were similar to the CM whist the last step consisted of homogenizing the gastric biopsy tissues using a BB (Next Advance, Averill Park, NY, USA) (Figure 1). To perform these methods, media was removed from the biopsies by using a 70-μm cell strainer (BD Falcon, Franklin Lakes, NJ, USA) and dried through the filter using sterile gauze. The tissue was then transferred to a pre-weighted 1.5 ml centrifuge tube and the net weight measured. Biopsies were then rapidly transferred to a 50 ml conical tube containing 10 ml of HBSS without CaCl_2_, MgCl_2_, MgSO_4_ (Gibco) with antibiotics/antifungal mix (Gibco) and EDTA (1 mM) and incubated at 37°C for 30 min while shaking. The tissues were washed with 10 ml of HBSS buffer (with CaCl_2_, MgCl_2_) (Gibco) without EDTA and incubated for 10 min at room temperature (RT) while shaking. The tissues were then enzymatically digested either in six well plates (CM method) or 1.5 ml sterile screw-top polypropylene microcentrifuge tubes (BB method) containing 1 ml of digestion solution. Tubes used for the BB method also contained two stainless steel beads (3.2 mm diameter; Next Advance Inc., Averill, NY, USA). The enzymatic digestion solution consisted of 1 ml of RPMI containing 10 μl of fetal bovine serum (FBS) (Gemini Bioproducts, West Sacramento, CA, USA), 10 μl antibiotics/antifungal mix (Gibco), 10 μl of 2.5 M CaCl_2_, 10 μl of Collagenase D (100 μg/ml; Roche, Indianapolis, IN, USA), and 1 μl DNase I (10 μg/ml; Affymetrix, Cleveland, OH, USA). The biopsies (20 mg maximum per tube) were digested for 45 min at 37°C with intermittent pipetting (CM method) or shaking (BB method). Following 45 min incubation, the tissues were disaggregated using the frosty ends of glass slides (CM method). In the case of the BB method, following the 45 min digestion the tube was placed in a BB homogenizer (Next Advance Inc., NY, USA) and the tissue homogenized for 30 s (speed 1). Tissues were further incubated for 15 min (37°C). After the second digestion by either method, cells were collected in a 50 ml tube through a 70 μm cell strainer and centrifuged at 1400 rpm. Cells were then washed and re-suspended in complete RPMI [Heat inactivated FBS (10%), l-glutamine (2 mM), non-essential Amino acids (1×) (Gibco 11140), HEPES buffer (10 mM) (Gibco 15630-080), Sodium pyruvate (2.5 mM) (Lonza 13-155E), Penicillin/streptomycin (100 U/ml–100 μg/ml) (Sigma P0781), Gentamicin (50 μg/ml) (Gibco 15750-060)], and counted using Kova Glasstic Slides (Hycor Biomedical, CA, USA). Cells were either stained immediately for immunophenotyping by flow cytometry or stimulated with mitogens overnight before staining (see below). To evaluate whether the enzymatic digestion resulted in loss of surface receptors, PBMC were either treated or untreated with the same digestion mix and processed as detailed above and assessed for surface markers (Figures S1C,D in Supplementary Material). To determine the effect of collagenase D in the digestion mix on surface marker expression the above procedure was followed using an enzymatic mix in which collagenase D was replaced with dispase (1 μg/ml) (Figure S1C in Supplementary Material).

### Flow cytometry procedures and staining

#### *Ex vivo* stimulation

Freshly isolated cells from PBMC and gastric biopsies (LPMC) were re-suspended in complete media and stimulated with medium, staphylococcal enterotoxin B (SEB) (10 μg/ml; Sigma) or Dynabeads Human T activator CD3/CD28 (4 × 10^4^ beads/ml) (Invitrogen Dynal, Oslo, Norway). For each treatment, 1 × 10^5^ LPMC and 1 × 10^6^ PBMC were cultured in 200 μl and 1 ml total volumes, respectively, and incubated at 37°C in 5% CO_2_. In some experiments, cells were stained with CD107a-FITC at the time of stimulation. After 2 h, GolgiStop (Monensin, BD) and GolgiPlug (Brefeldin A, BD) were added at concentrations of 0.5 μl/ml and cultures continued overnight at 37°C in 5% CO_2_.

#### Surface and intracellular staining

Following stimulation, PBMC and LPMC were plated in 96-well V-bottom plates for staining. Cells were washed twice with phosphate buffered saline (PBS) and stained for live/dead discrimination using Invitrogen LIVE/DEAD fixable yellow dead cell stain kit (YEVID) (Invitrogen, Carlsbad, CA, USA). Blocking of Fc receptors was performed using human immunoglobulin (3 μg/ml; Sigma) and was followed by surface staining, performed as previously described (21). Briefly, cells were stained with fluorescently labeled monoclonal antibodies (mAbs) directed to CD14-BV570 (clone M5E2, Biolegend, San Diego, CA, USA) and CD19-BV570 (clone HIB19, Biolegend), CD3-BV650 (clone OKT3, Biolegend), CD4-PE-Cy5 (clone RPA-T4, BD), CD8-PerCP-Cy5.5 (clone SK-1, Becton–Dickinson, BD), CD45RA-biotin (clone HI100, BD), integrin α4β7-Alexa Fluor 647 (clone Act-1, Leukosite, Cambridge, MA, USA), and CD62L-Alexa Fluor 780 (clone DREG-5, eBioscience, San Diego, CA, USA) at 4°C for 30 min. Staining with streptavidin-QDot800 (Invitrogen) was performed for panels that included biotin-conjugated mAbs for 30 min at 4°C. The cells were then fixed and permeabilized using IC fixation and permeabilization buffers (eBioscience) according to the manufacturer’s recommendations. Intracellular staining with mAbs to IL-17A-BV421 (clone BL168, Biolegend), IL-2-BV605 (clone MQ1-17H12, Biolegend), IFN-γ-PE-Cy7 (clone B27, BD), TNF-α-Alexa 700 (clone MAb11, BD), MIP-1β-PE (clone 24006, R&D Systems, Minneapolis, MN, USA), and CD69-ECD (clone TP1.55.3, Beckman Coulter, Danvers, MA, USA) was performed at 4°C overnight. After staining, cells were fixed in 1% paraformaldehyde and stored at 4°C until data collection. Data were collected using a customized LSRII flow cytometer (BD) and then analyzed using WinList version 7 (Verity Software House, Topsham, ME, USA) software package. Graphs were generated using GraphPad Prism version 5.03 (GraphPad Software, San Diego, CA, USA).

In experiments designed to characterize T_RM_ cells, the staining panels were modified as follows. LPMC and PBMC were stained with mAbs directed to CD103-Alexa Fluor 488 (clone Ber-ACT8, Biolegend), CD14-BV570 (clone M5E2, Biolegend, San Diego, CA, USA), CD13-Pacific Orange (clone WM-15 eBioscience, San Diego, CA, USA conjugated to Pacific Orange in-house), CD19-BV570 (clone HIB19, Biolegend), CD3-BV650 (OKT3, Biolegend), CD4-PE-Cy5 (clone RPA-T4, BD), CD8-PerCP-Cy5.5 (clone SK-1, BD), CD45RA-biotin (clone HI100, BD), integrin α4β7-Alexa Fluor 647 (clone Act-1, Leukosite, Cambridge, MA, USA), and CD62L-Alexa Fluor 780 (clone DREG-5, eBioscience, San Diego, CA, USA) and intracellularly with mAbs to IL-17A-BV421 (clone BL168, Biolegend), IL-2-BV605 (clone MQ1-17H12, Biolegend), IFN-γ-PE-Cy7 (clone B27, BD), TNF-α-Alexa 700 (clone MAb11, BD), MIP-1β-PE (clone 24006, R&D systems, Minneapolis, MN, USA), and CD69-ECD (clone TP1.55.3, Beckman Coulter, Danvers, MA, USA).

### FCOM analysis for multifunctionality

FCOM (Verity Software House, Topsham, ME, USA) is an analytical tool that is used to classify events based on combinations of selected gates. FCOM reduces multiparameter data to a series of multiple acquisition gates, one for every possible combination. FCOM was employed to determine the subsets of CD4^+^ and CD8^+^ producing multiple cytokines and/or expressing CD107a expression (i.e., multifunctionality).

### Statistical analysis

Data were analyzed using GRAPHPAD PRISM™ 5.03 statistical software (Graphpad, San Diego, CA, USA). Statistical differences in median values between two groups were determined using Mann–Whitney tests. Statistical differences between multiple groups (more than two) were determined by Kruskal–Wallis tests and the Dunn’s post-test was used to compare selected group pairs. Values of *p < 0.05, **p < 0.005, ***p < 0.0005 were considered significant.

## Results

### Gastric LPMC isolation and cell yields from children, adults, and elderly volunteers

Several methodologies to isolate gastric leukocytes from human stomach biopsies have been reported; however, there is a lack of consensus in the type of digestion enzymes to use, their concentration, digestion periods and whether or not to use mechanical dissociation techniques. Therefore, we optimized a protocol for isolation of gastric LPMC. We first compared two methods: (i) a conventional method (CM) and (ii) a blender method (BB) (described in detail in Section “Materials and Methods”). In the BB method, we optimized the homogenization step regarding the speed, time, and number of beads needed for a gentle dissociation of the cells from the gastric tissues. We found that homogenizing the tissue for 30 s at a speed of 1 and using 2 beads (stainless steel; 3.2 mm diameter) resulted in optimal cell yields (Figure S1A in Supplementary Material). This optimized BB method yielded superior cell numbers (1.1 × 10^4^/mg of tissue) from human biopsies compared to the CM method (0.6 × 10^4^/mg of tissue) (Figure S1B in Supplementary Material). Two digestion enzymes (collagenase D and dispase) were then compared by substituting each one using the optimized BB method. We observed that collagenase D treatment resulted in better cell yields and cell surface marker preservation than dispase, which had a marked effect on the expression of cell surface markers as shown by lower MFI for CD4^+^ and CD8^+^ T cells in both PBMC and LPMC isolated cells (Figure S1C in Supplementary Material). We further evaluated the effect of collagenase D on tissues by using PBMC treated in similar fashion as biopsies with the BB method. The results showed no significant differences in the expression levels of CD3, CD4, CD8, CD45RA, CD62L, and integrin α4β7 surface markers between PBMC treated with or without collagenase D (Figure S1D in Supplementary Material).

**Figure 2:**
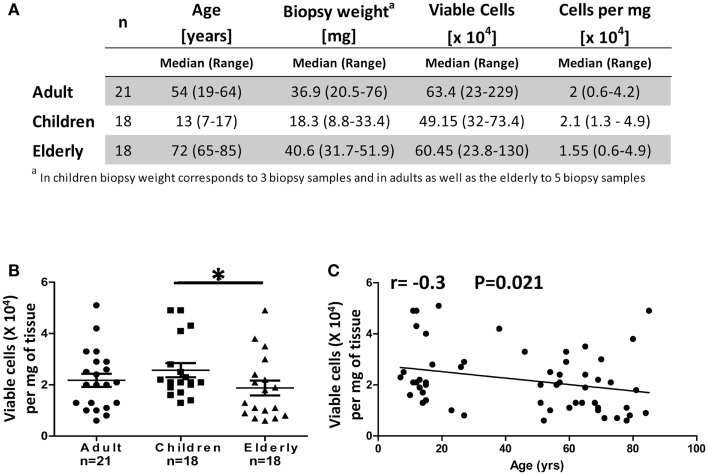
**Cell yields from gastric biopsies (LPMC) from different age groups**. **(A)** Comparison of biopsy weight and viable cell yields obtained from children, adults, and the elderly. Trypan blue exclusion was used to enumerate live viable cells. **(B)** Cell yields were expressed as total viable cells per milligram of tissue and compared between the three age groups. **(C)** Correlation between the age of volunteers and cell yields obtained from biopsies as continuous variables. Results were analyzed using Spearman’s correlation (*n* = 57); *p < 0.05.

Stomach biopsies (antrum) obtained from *H. pylori* negative (CLO test negative) adult (18–64 years), children (7–17 years), and elderly (65–85 years) volunteers were processed as described in Section “Materials and Methods” and Figure 1. Gastric LPMC were isolated and enumerated from five biopsy samples from each adult and elderly volunteer and three biopsy samples from each child (Figure 2A). The viable cell yields in biopsies from adult and the elderly ranged from 230,000 to 2,300,000 (median 634,000) and 240,000 to 1,300,000 (median 605,000) cells, respectively; whereas in children’s biopsies cell yields ranged from 320,000 to 734,000 (median: 492,000) cells (Figure 2A). The total viable cell yields in the children group was significantly lower (*p* < 0.05) than in the adult group. However, the weight of biopsies varied between age groups as samples from children were significantly (*p* < 0.05) smaller in size and weight (8.8–33.4 mg) than samples from adults (20.5–76 mg) and the elderly (31.7–51.9 mg) (Figure 2A). To compare the viable cell yields among age groups we calculated the number of viable cells per milligram of tissue. The results showed that the cell yields obtained from biopsies of children (13,000–49,000 viable cells/mg of tissue, median: 21,000) were significantly higher (*p* < 0.05) than those obtained from biopsies of the elderly (6000–49,000, median: 15,500) (Figures 2A,B). Finally, using Pearson’s regression analysis, we observed a significant inverse correlation (*r* = −0.3, *p* = 0.021) between the number of isolated cells per milligram of tissue and age (Figure 2C).

### Characterization of T cell subsets in LPMC and PBMC

**Figure 3:**
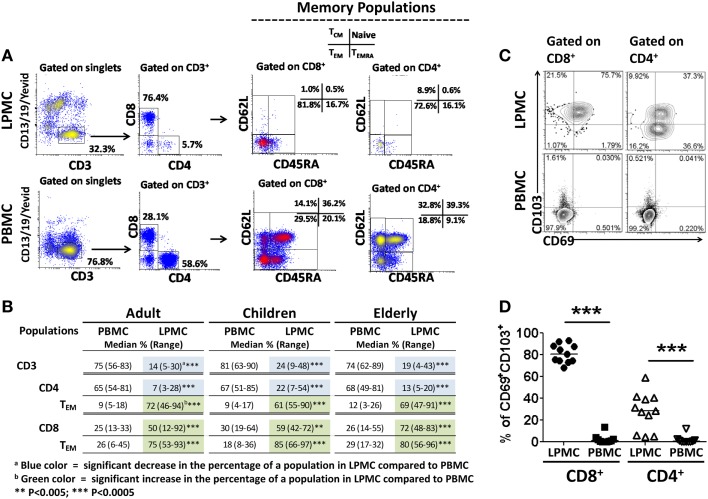
**Characterization of memory T (T_M_) cells in gastric LPMC and PBMC from children, adults, and the elderly**. **(A)** Representative scatter plots of the gating strategy used to characterize T cell subsets in LPMC and PBMC: naïve (CD62L^+^CD45RA^+^), central memory (T_CM_, CD62L^+^CD45RA^−^), effector memory (T_EM_, CD62L^−^CD45RA^−^), and effector memory expressing CD45RA (T_EMRA_, CD62L^−^CD45RA^+^). Data shown are from a 12 year-old child. **(B)** Cumulative data showing the median % and range of CD3, CD4, CD8, T_EM_ populations in gastric LPMC and PBMC from all three age groups. **(C)** Identification of tissue-resident memory T (T_RM_) cells in gastric LPMC. Representative plots showing expression of T_RM_ cells as defined by the concomitant expression of CD103 and CD69 markers on CD8^+^ and CD4^+^ T cells in gastric LPMC and PBMC. **(D)** Cumulative data (*n* = 10) showing the percentage of gastric T_RM_ cells among CD8^+^ and CD4^+^ T cells from LPMC and PBMC (***p < 0.0005).

**Figure 4:**
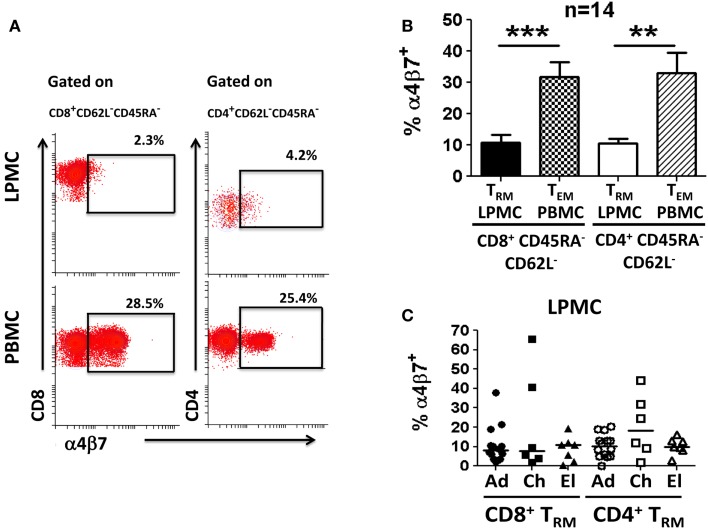
**Characterization of integrin α4β7 expression on memory T (T_RM_ and T_EM_) cells in gastric LPMC and PBMC**. **(A)** Representative scatter plots showing integrin α4β7 expression on CD8^+^ and CD4^+^ gastric LPMC and PBMC (data shown are from a 12-year-old child). **(B)** Cumulative data (*n* = 14) showing the percentage of CD8^+^ and CD4^+^ T_RM_ (LPMC) as well as CD8^+^ and CD4^+^ T_EM_ (PBMC) cells expressing integrin α4β7. Significant differences are denoted as follows: **p < 0.005; ***p < 0.0005. **(C)** Cumulative data comparing the percentage of CD8^+^ or CD4^+^ T_RM_ subsets (LPMC) expressing integrin α4β7 by age group. Closed and open symbols represent CD8^+^ and CD4^+^ T_RM_ cells, respectively: adults (Ad); children (Ch); elderly (El).

Human gastric T lymphocytes have been shown to display a Th1 type response (IFN-γ, TNF-α, and IL-2 secretion) toward pathogens, such as *H. pylori* (16). However, the T_M_ subset(s) that secrete(s) these cytokines and differences among the various age groups have not been explored. To address these shortcomings, the presence of T cells (CD3^+^CD13^−^CD19^−^) in LPMC and PBMC was assessed (Figure 3A). The frequency of these cells was significantly lower in LPMC than in PBMC from adult volunteers (median: 13.7 vs. 74.7%; *p* < 0.0005) (Figures 3A,B). Similar findings were observed in children and the elderly (Figure 3B). When CD3^+^ T cells were divided into CD4^+^ (CD3^+^CD4^+^) and CD8^+^ (CD3^+^CD8^+^) T cells, the latter were more abundant in LPMC than in PBMC (Figures 3A,B). Therefore, there was an inversion in the CD4/CD8 ratio in LPMC (~1:3) compared to PBMC (~3:1). These results confirmed and extended those reported by others (16, 19, 20) by showing that these differences were observed in all age groups (Figure 3B). When CD4^+^ T cells in LPMC of children and the elderly were compared to adults, children showed a significantly higher frequency of these cells (Figure 3B). In contrast, PBMC from all three groups expressed similar levels of CD3^+^, CD4^+^, and CD8^+^ T cells (Figure 3B). Next, we evaluated the presence of T_M_ cells in LPMC and PBMC using CD62L and CD45RA markers. These markers define four different subsets: (i) T central memory (T_CM_) (CD62L^+^CD45RA^−^), (ii) T naïve (T_naive_) (CD62L^+^CD45RA^+^), (iii) T T_EM_ (CD62L^−^CD45RA^−^), and (iv) CD45RA positive T T_EMRA_ (CD62L^−^CD45RA^+^) (22). Interestingly, the vast majority of adult CD8^+^ and CD4^+^ T cells in LPMC showed a T_EM_ phenotype (>70%) (CD62L^−^, CD45RA^−^) (Figures 3A,B). This phenotype was also dominant in children and the elderly (Figure 3B). In contrast, the classic memory (T_CM_, T_EM_, and T_EMRA_) and naïve subsets defined by CD62L and CD45RA were identified in PBMC (Figures 3A,B). The finding that LPMC CD4^+^ and CD8^+^ T cells showed a dominant T_EM_ phenotype suggested that these cells could represent a population of gastric tissue-resident memory T (T_RM_) cells. The hallmark of T_RM_ is the surface expression of high levels of CD103 and CD69 (4, 6–8); therefore, we evaluated expression of these markers in LPMC and PBMC. We observed that most CD8^+^ T cells in LPMC co-expressed CD103 and CD69 (mean: 81.1%; median: 80.5%), while a much lower percentage of CD4^+^ T cells in LPMC co-expressed these markers (mean: 35%; median: 28.8%) (Figures 3C,D). Similar results were observed in all the volunteers tested (Figure 3D). On the other hand, CD103 and CD69 were virtually absent from CD4^+^ and CD8^+^ T cells in PBMC. Further analysis revealed that CD8^+^ T cells in LPMC either co-expressed CD103 and CD69 (>70%) or expressed CD103 alone (~20%); therefore, more than 90% of these cells expressed at least one marker that defined them as T_RM_ cells (Figure 3C). Similarly, even though only ~30% of CD4^+^ T cells in LPMC co-expressed CD103 and CD69, expression of CD69 or CD103 alone was found in ~35 and ~10% of these cells, respectively. In sum, the majority of the CD4^+^ and CD8^+^ T cells in LPMC expressed molecules compatible with those reported for T_RM_ cells. In contrast, the expression of CD103 and CD69 was virtually absent from PBMC CD4^+^ and CD8^+^ T cells (Figures 3C,D). Expression of the homing marker integrin α4β7 was assessed in PBMC and LPMC in the subsets showing the T_EM_ phenotype (T_RM_ in LPMC and T_EM_ in PBMC) (Figure 4A). Gastric CD8^+^ T_RM_ and CD4^+^ T_RM_ showed a significantly lower level of expression of integrin α4β7 (*p* < 0.05) compared to CD8^+^ T_EM_ and CD4^+^ T_EM_ (Figures 4A,B). Interestingly, there were no significant differences in integrin α4β7 expression among gastric LPMC CD8^+^ T_RM_ or CD4^+^ T_RM_ between adult, children, and the elderly (Figure 4C).

### Mitogen activation of LPMC and PBMC

**Figure 5:**
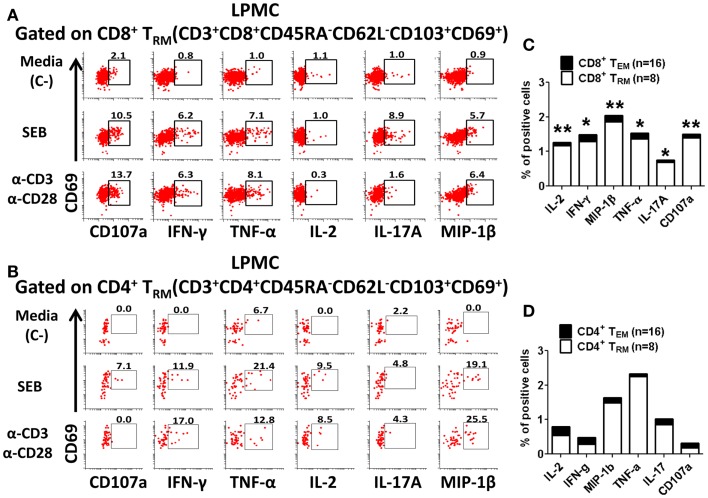
**Activation of CD8^+^ and CD4^+^ tissue-resident memory T (T_RM_) cells in gastric LPMC**. Representative plot of the activation of gastric LPMC CD8^+^ **(A)** or CD4^+^ **(B)** T_RM_ (CD62L^−^CD45RA^−^CD69^+^ CD103^+^) by two stimulants: (1) staphylococcal enterotoxin B (SEB; 10 μg/ml) and (2) anti-CD3/CD28 beads (α-CD3 α-CD28) to produce IL-2, IFN-γ, MIP-1β, TNF-α, IL-17A, and up-regulation of the expression of CD107a. Cells left unstimulated were used as negative control (Media, C-). Cumulative data comparing baseline activation levels of gastric LPMC CD8^+^ **(C)** and CD4^+^ **(D)** T_RM_ (white portion of the bar) to PBMC (black portion of the bar). In **(C,D)** significant differences between T_EM_ and T_RM_ are indicated with asterisks on top of each bar; *p < 0.05; **p < 0.005; ***p < 0.0005.

We next examined whether isolated gastric LPMC CD8^+^ or CD4^+^ T_RM_ cells were functionally active by exploring whether they responded to mitogen stimulation by producing cytokines and up-regulating the expression of CD107a, a marker of degranulation associated with cytotoxic activity (23). Furthermore, we explored whether there were any differences in the responses between the different age groups. Gastric LPMC and PBMC were stimulated with various T cell stimulants including: (i) SEB (superantigen) and (ii) α-CD3/CD28 coated beads (TCR stimulation). As negative control, cells were incubated in media alone. The concomitant production of multiple cytokines (IFN-γ, TNF-α, IL-2, IL-17A, and MIP-1β) and up-regulation of CD107a were determined in LPMC (Figures 5A,B) and PBMC in all three age groups. Up-regulation of CD69 as a cell activation marker was only considered for PBMC since in LPMC (T_RM_) this marker is highly expressed regardless of stimulation (Figures 3C,D). In PBMC, the main T_M_ subsets responding to the stimulations were CD8^+^ and CD4^+^ T_EM_ and T_EMRA_ and these results were consistent with previous reports from our group, as well as others (6, 24). The percentages of gastric CD8^+^ T_RM_ and CD4^+^ T_RM_ producing cytokines and up-regulating CD107a following stimulation were compared to CD8^+^ T_EM_ and CD4^+^ T_EM_ (PBMC) and the results in all age groups are summarized in Tables 1–4.

**Table 1:** **Comparison of PBMC CD8^+^ T_EM_ and gastric LPMC CD8^+^ T_RM_ cells activation responses in adult, children, and the elderly**.

Cytokine	Stimulant	Adult		Children		Elderly	
		CD8^+^ T_EM_	CD8^+^ T_RM_	CD8^+^ T_EM_	CD8^+^ T_RM_	CD8^+^ T_EM_	CD8^+^ T_RM_
		Median	% (Range)	Median	% (Range)	Median	% (Range)
IL-2	Media	0.1 (0– 2)	1.2 (0– 2)^a,^**	0.3 (0–1)	1.0 (0–2)	0.1 (0– 1)	1.7 (0– 3)**
	SEB	1.7 (0– 17)	1.6 (0– 42)	0.3 (0– 2)	1.5 (1– 2)	1.8 (1– 13)	3.9 (1– 22)
	α-CD3/CD28	2.0 (0– 12)	1.7 (0– 11)	0.3 (0– 1)	1.0 (0– 3)	1.4 (1– 9)	4.0 (1– 12)
INF-γ	Media	0.2 (0– 6)	1.3 (0– 3)*	0.4 (0– 3)	1.6 (1– 3)	0.6 (0– 2)	1.7 (0– 5)
	SEB	8.6 (0– 37)	15.7 (8– 27)	4.3 (1– 19)	10.3 (6– 17)	6.3 (2– 57)	11.5 (2– 28)
	α-CD3/CD28	3.1 (0– 19)	13.8 (3– 30)**	2.7 (1– 6)	12.3 (6– 23)*	6.5 (1– 15)	20.9 (10– 28)*
MIP-1β	Media	0.2 (0– 6)	1.9 (0– 8)**	0.5 (0– 4)	0.6 (0– 6)	1.0 (0– 4)	4.8 (1– 10)*
	SEB	5.0 (0– 20)	15.6 (5– 31)*	4.4 (0– 7)	8.6 (3– 15)	15.5 (1– 55)	17.8 (3– 30)
	α-CD3/CD28	2.3 (0– 20)	16.7 (3– 49)**	1.4 (0– 14)	8.9 (2– 42)	14.6 (1– 39)	33.2 (8– 55)
TNF-α	Media	0.2 (0– 3)	1.4 (0– 4)*	0.9 (0– 3)	1.9 (1– 3)	0.9 (0– 2)	0.7 (0– 4)
	SEB	2.6 (0– 32)	9.1 (4– 22)	2.6 (2– 21)	3.4 (1– 15)	9.4 (1– 49)	7.6 (1– 17)
	α-CD3/CD28	2.9 (0– 13)	7.8 (2– 15)*	1.9 (1– 5)	8.5 (4– 11)	9.9 (1– 21)	9.7 (1– 13)
IL-17A	Media	0.1 (0– 2)	0.7 (0– 2)*	0.5 (0– 1)	1.1 (0– 2)	0.1 (0– 1)	0.8 (0– 2)
	SEB	1.1 (0– 4)	1.1 (0– 7)	0.3 (0– 2)	2.2 (0– 9)	0.2 (0– 1)	0.4 (0– 10)
	α-CD3/CD28	2.0 (0– 5)	1.0 (0– 3)	0.2 (0– 1)	1.9 (0– 2)*	0.2 (0– 1)	0.9 (0– 5)**
CD107a	Media	0.1 (0– 4)	1.4 (0– 3)**	0.8 (0– 4)	3.4 (2– 4)	1.1 (0– 4)	2.6 (1– 5)
	SEB	5.2 (0– 21)	12.9 (7– 40)*	4.6 (3– 21)	9.8 (7– 23)	14.2 (5– 45)	12.9 (3– 26)
	α-CD3/CD28	1.3 (0– 12)	10.8 (2– 18)**	3.0 (2– 7)	16.6 (7– 28)**	12.7 (1– 21)	12.3 (10– 19)

*Gastric LPMC CD8^+^ T_RM_ responses (IL-2, IFN-γ, MIP-1β, TNF-α, IL-17A, and CD107a) to two stimulants (SEB and α-CD3/CD28 beads) were compared to PBMC CD8^+^ T_EM_ responses obtained from adults, children, and the elderly. Significant differences are shown in highlighted colors as determined by Mann–Whitney tests. Adults: *n* = 10; children: *n* = 10; elderly: *n* = 10*.

^a^ Light green color = significant increase in the frequency of CD8^+^ T_rm_ (LPMC) compared to CD8^+^ T_em_ (PBMC); *p < 0.05; **p < 0.005.

Interestingly, control media gastric CD8^+^ and CD4^+^ T_RM_ cells from adult volunteers showed higher percentages of cells producing cytokines and up-regulating expression of CD107a than media only CD8^+^ T_EM_ and CD4^+^ T_EM_ (PBMC) cells (Figures 5C,D). This difference in baseline cytokine production was statistically significant (*p* < 0.05) only in CD8^+^ T cells (Figures 5C,D). Of note, in children, while higher CD8^+^ T_RM_ cells producing cytokines and up-regulating the expression of CD107a were observed, no statistically significant differences were found compared to CD8^+^ T_EM_ cells in media controls (Table 1). In the elderly group, a percentage of cells producing significant higher baseline levels of IL-2 and MIP-1β in gastric CD8^+^ T_RM_ cells were identified. Concerning the other cytokines investigated, as well as CD107a, although a similar trend to the other age groups was observed, the differences did not reach statistical significance, likely due to high subject-to-subject variability (Table 1). Additionally, the diagnostic pathology reports allowed us to explore whether the higher baseline cytokines levels of CD8^+^ and CD4^+^ T_RM_ cells were due to extrinsic factors causing inflammation of the antral mucosa. Volunteers were classified as having either normal or mildly inflamed (mild diffuse erythema, mild diffuse inflammation, reactive changes) antral mucosa and the baseline cytokines levels were compared between these two groups (Figure S2 in Supplementary Material). There were no differences between the normal and “mild inflammation” groups for any of the cytokines/chemokines (IL-2, IFNγ, MIP-1β, TNFα, and IL-17A) and CD107a at baseline in CD8^+^ T_RM_ cells (Figure S2 in Supplementary Material). Although CD4^+^ T_RM_ cells showed somewhat increased baseline cytokines levels (IL-2, IFNγ, TNFα, and IL-17A) in the “mild inflammation” group, they were not statistically different than those observed in the normal group (Figure S2 in Supplementary Material).

Gastric CD8^+^ T_RM_ and CD4^+^ T_RM_ cells from adult volunteers stimulated with mitogens (SEB and anti-CD3/CD28) produced most of the assessed cytokines at higher levels than media control cells (Figures 5A,B; Tables 1–4). In general, higher percentages were observed in gastric CD8^+^ T_RM_ and CD4^+^ T_RM_ cells producing cytokines and expressing CD107a than in CD8^+^ T_EM_ and CD4^+^ T_EM_ cells. In some instances, the cytokine production differences were statistically significant (Tables 1 and 2). Overall, results were similar in all age groups (Tables 1 and 2).

**Table 2:** **Comparison of PBMC CD4^+^ T_EM_ and gastric CD4^+^ T_RM_ cells activation responses in adult, children, and the elderly**.

Cytokine	Stimulant	Adult		Children		Elderly	
		CD4^+^ T_EM_	CD4^+^ T_RM_	CD4^+^ T_EM_	CD4^+^ T_RM_	CD4^+^ T_EM_	CD4^+^ T_RM_
		Median	% (Range)	Median	% (Range)	Median	% (Range)
IL-2	Media	0.3 (0– 3)	0.5 (0– 2)	0.5 (0– 2)	1.3 (0– 3)	0.3 (0– 4)	1.6 (0– 9)
	SEB	2.5 (0– 60)	3.4 (0– 35)	1.5 (1– 5)	0 (0– 6)	14.8 (1– 31)	8.7 (0– 32)
	α-CD3/CD28	2.2 (0– 38)	8.9 (0– 21)	1.2 (0– 6)	8.8 (0– 20)	7.9 (3– 27)	21.4 (5– 32)
INF-γ	Media	0.2 (0– 2)	0.3 (0– 2)	0.7 (0– 3)	4.8 (0– 9)	0.6 (0– 3)	0.0 (0– 5)
	SEB	3.0 (0– 15)	7.0 (0– 11)	5.8 (2– 9)	3.7 (0– 25)	6.2 (3– 18)	3.6 (1– 19)
	α-CD3/CD28	0.6 (0– 5)	5.6 (5–22)^a^,***	4.1 (1–9)	10.9 (2–25)	3.8 (1– 14)	14.1 (2– 27)
MIP-1β	Media	0.2 (0– 1)	1.5 (0– 8)	0.3 (0– 2)	4.7 (0– 6)	0.7 (0– 1)	1.3 (0– 8)
	SEB	1.5 (0– 13)	4.8 (−33)	0.8 (0– 5)	11.4 (0– 35)	1.5 (1– 3)	13.5 (1–21)*
	α-CD3/CD28	0.8 (0– 9)	9.2 (1– 38)**	0.3 (0– 3)	15.0 (0– 31)	1.2 (0– 3)	0.0.3 (7– 34)***
TNF-α	Media	0.1 (0– 3)	2.3 (0– 6)	1.2 (0– 6)	4.2 (1– 6)	2.8 (0– 5)	1.2 (0– 6)
	SEB	0.9 (0– 55)	10.8 (2– 30)	15.7 (5– 21)	6.3 (2– 14)	41.1 (1– 53)	9.9 (0–21)^b,^*
	α-CD3/CD28	0.6 (24)	5.8 (14– 42)***	12.2 (5– 25)	24.6 (8– 60)	23.7 (2– 31)	27.6 (4– 33)
IL-17A	Media	0.2 (0– 2)	0.9 (0– 3)	0.3 (0– 3)	1.4 (0– 3)	0.3 (0– 1)	0.0 (0– 3)
	SEB	2.1 (1– 16)	2.4 (0– 12)	3.1 (0– 7)	6.3 (2– 25)	1.5 (1– 3)	2.1 (1– 9)
	α-C03/CD28	3.8 (0– 14)	4.8 (0– 12)	1.6 (1– 5)	14.1 (3–19)*	0.9 (0– 2)	2.9 (0– 14)
CD107a	Media	0.2 (0– 1)	0.2 (0– 3)	0.3 (0– 1)	1.3 (0– 5)	0.2 (0– 2)	0.0 (0– 1)
	SEB	1.5 (1– 7)	4.0 (0– 11)	1.2 (0– 3)	5.7 (2–19)*	1.6 (1– 3)	2.2 (0– 11)
	α-C03/C028	0.7 (0– 3)	5.9 (0– 8)*	0.7 (0– 2)	6.3 (2–12)*	1.4 (0– 3)	3.5 (0– 12)

*Gastric LPMC CD4^+^ T_RM_ responses (IL-2, IFN-γ, MIP-1β, TNF-α, IL-17A, and CD107a) to two stimulants (SEB and anti-CD3/CD28) were compared to PBMC CD4^+^ T_EM_ responses obtained from adults, children, and the elderly. Significant differences are shown in highlighted colors as determined by Mann–Whitney tests. Adults: *n* = 10; children: *n* = 10; elderly: *n* = 10*.

*^a^ Light green color = significant increase in the frequency of CD4^+^ T_rm_ (LPMC) compared to CD4^+^ T_em_ (PBMC)*.

^b^ Light blue color = significant decrease in the frequency of CD4^+^ T_rm_ (LPMC) compared to CD4^+^ T_em_ (PBMC); *p < 0.05; **p < 0.005; ***p < 0.0005.

We next compared cytokine production by adults, children, and the elderly in both PBMC and LPMC populations (results are summarized in Table 3). CD8^+^ T_EM_ cells from children did not show statistically significant differences when compared to adults for any of the cytokines evaluated or CD107a expression. In contrast the elderly group demonstrated significantly higher number of CD8^+^ T_EM_ cells (*p* < 0.05) producing MIP-1β at baseline levels (compared to adults) and this cytokine was identified in a higher percentage of cells following stimulation (Table 3). Other cytokines observed in a higher percentage of cells following stimulation in the elderly were TNF-α (anti-CD3/CD28) and CD107a expression (SEB and anti-CD3/CD28) (Table 3). In children, a significantly higher percentage of CD8^+^ T_RM_ cells (LPMC) expressed CD107a at baseline than in adults (Table 3). Neither children nor the elderly showed differences in the percentage of CD4^+^ T_EM_ cells (PBMC) producing cytokines compared to adults. On the other hand, in children, at baseline, the percentage of CD4^+^ T_RM_ cells (LPMC) producing IFN-γ and TNF-α was higher than in adults. However, no differences were noted following stimulation (Table 3). In the elderly, the percentage of CD4^+^ T_RM_ cells producing TNF-α, at basal levels were also significantly higher than in adults. Moreover, the percentage of CD4^+^ T_RM_ cells producing significantly higher levels of IL-2 (anti-CD3/CD28), IFN-γ (anti-CD3/CD28), and TNF-α (SEB and anti-CD3/CD28) following stimulation was also enhanced (Table 3).

**Table 3:** **Summary of gastric LPMC T_RM_ cells and PBMC T_EM_ (CD4^+^ and CD8^+^) responses between children and the elderly to adults**.

Cytokine	Stim	PBMC		LPMC		PBMC		LPMC	
		CD8^+^ T_EM_		CDS^+^ T_RM_		CD4^+^ T_EM_		CD4^+^ T_RM_	
		Ch^a^	El^b^	Ch	El	Ch	El	Ch	El
IL-2	Med								
	SEB								
	α-CD3/CD28^c^								*
IFN-γ	Med							*	
	SEB								
	α-CD3/CD28								*
MIP-1β	Med		*^,d^						
	SEB		*						
	α-CD3/CD28		*						
TNF-α	Med							*	*
	SEB								**
	α-CD3/CD28		*						**
IL-17A	Med								
	SEB								
	α-CD3/CD28								
CD107a	Med			*					
	SEB		*						
	α-CD3/CD28		**						

*LPMC CD8^+^ and CD4^+^ T_RM_ and PBMC T_EM_ responses (IL-2, IFN-γ, MIP-1β, TNF-α, IL-17A, and CD107a) to stimulation by two mitogens (SEB and anti-CD3/CD28) were determined and compared between children (Ch) or elderly (El) to adults (children vs. adult and elderly vs. adult). Significant differences are shown in highlighted colors as determined by Mann–Whitney test. Adults: *n* = 8; children: *n* = 5; elderly: *n* = 7. Empty (white) cells indicate non-significant differences between children or elderly vs. adults as determined by Mann–Whitney tests*.

^a^ Children; ^b^Elderly; ^c^Anti-CD3/CD28 beads; ^d^Light green color = significant in cytokine production compared to *p < 0.05; *p < 0.005.

**Table 4:** **Summary of T_RM_ and T_EM_ responses to stimulation in children, elderly, and adults**.

Cytokine	Age	PBMC	CD8^+^ T_EM_	LPMC	CD8^+^ T_RM_	PBMC	CD4^+^ T_EM_	LPMC	CD48^+^ T_RM_
				
		SEB	α3/28^a^	SEB	α3/28	SEB	α3/28	SEB	α3/28
IL-2	Ad^b^	**^,e^	**			**	*		**
	Ch^c^								
	El^d^	***	**	*	*	**	*		*
IFN-γ	Ad	***	**	***	***	**	*	*	**
	Ch			**	**				*
	El	**	**	*	**	**	**	*	**
MIP-1β	Ad	**	*	**	***	**	*		*
	Ch								
	El	**	**	*	**	***	*	*	**
TNF-α	Ad	***	**	***	***	*	*	**	***
	Ch				**	*	*		**
	El	**	**		**	*	**	*	**
IL-17A	Ad	*	*			**	**		
	Ch							*	*
	El	*				**	**	*	
CD107a	Ad	***	***	***	**	***	***	*	**
	Ch	*		**	**				
	El	***	**	**	***	**	*	**	

*Gastric LPMC CD8^+^ and CD4^+^ T_RM_ and PBMC T_EM_ responses (IL-2, IFN-γ, MIP-1β, TNF-α, IL-17A, and CD107a) to stimulation by two mitogens (SEB and anti-CD3/CD28) were determined and compared to media stimulation (negative control) in adults (Ad), children (Ch), and the elderly (El). Significant differences are shown in highlighted colors as determined by Mann–Whitney tests. Adults: *n* = 8; children: *n* = 5; elderly: *n* = 7. Empty (white) cells indicate non-significant differences between stimulated and non-stimulated (media) cultures as determined by Mann–Whitney tests*.

^a^ Anti-CD3/CD28 beads; ^b^Adults; ^c^Children; ^d^Elderly; ^e^Light green color = significant increase in cytokine production compared to media stimulation, *p < 0.05; **p < 0.005; ***p < 0.0005.

We also compared the percentage of cells producing cytokines following SEB and anti-CD3/CD28 stimulation to the control cells in all age groups. The results are summarized in Table 4. SEB and anti-CD3/CD28 beads efficiently induced CD8^+^ T_EM_ and CD4^+^ T_EM_ cells from adults and the elderly to produce cytokines and CD107a expression compared to media control (Table 4). Interestingly, even though both stimulants induced CD8^+^ T_EM_ cells to produce cytokines in children (Table 1), the results were not statistically significant as compared to media (Table 4), and only CD107a up-regulation was significantly induced by SEB in children. SEB and anti-CD3/CD28 beads were unable to stimulate CD8^+^ T_RM_ cells to produce IL-17A in any of the three age groups and IL-2 was significantly induced only in the elderly (Table 4). CD8^+^ T_RM_ cells were efficiently induced to produce IFN-γ and CD107a expression in all three age groups by both SEB and anti-CD3/CD28 beads (*p* < 0.05). Furthermore, MIP-1β was efficiently induced in adults and the elderly, but not in children by both stimulants. TNF-α was also significantly induced in all three age groups but only by anti-CD3/CD28 beads. Neither SEB nor anti-CD3/CD28 beads were able to increase the percentage of CD4^+^ T_RM_ cells producing IL-2, MIP-1β, and expressing CD107a in children and IL-17A in adults. However, at least one of these stimulants was able to stimulate CD4^+^ T_RM_ to produce IL-2, IFN-γ, MIP-1β, TNF-α, and CD107a in adults and the elderly (Table 4).

### Multifunctional gastric CD8^+^ and CD4^+^ T_RM/EM_

**Figure 6:**
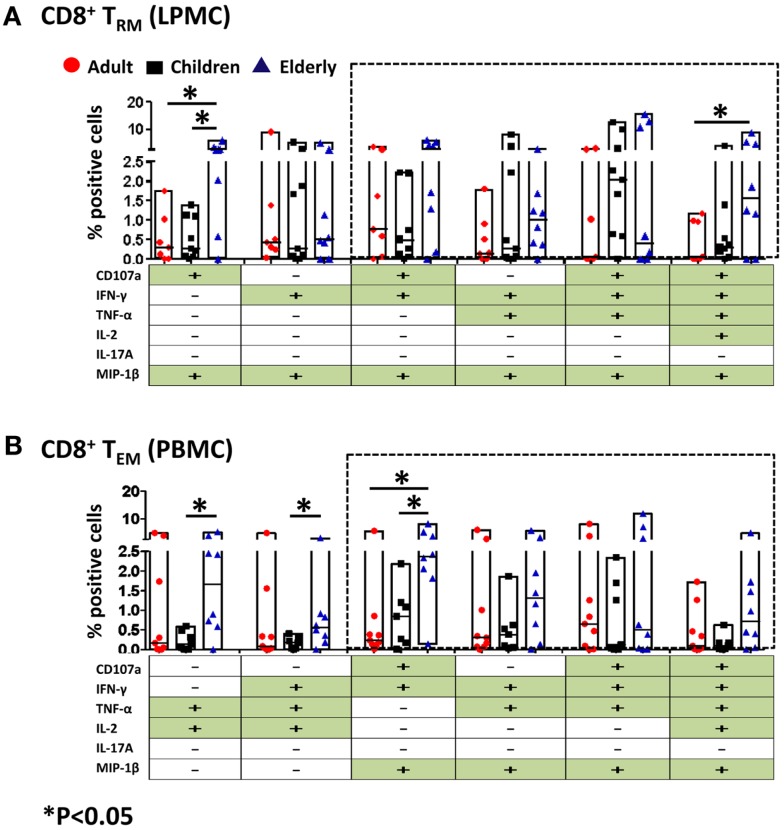
**Multifunctional gastric LPMC CD8^+^ T_RM_ and PBMC CD8^+^ T_EM_ responses to SEB stimulation in adults, children, and the elderly**. Multifunctionality was determined by simultaneous detection of two or more functions performed by CD8^+^ T_RM_ (LPMC) or CD8^+^ T_EM_ (PBMC). Six functions were evaluated: production of five cytokines/chemokines (IFN-γ, TNF-α, IL-2, IL-17A, MIP-1β) and expression of CD107a in response to SEB stimulation. **(A)** Scatter plot showing the six predominant function patterns in LPMC CD8^+^ T_RM_ and **(B)** in PBMC CD8^+^ T_EM_ cells from adults (red circles, *n* = 9), children (black squares, *n* = 7), and the elderly (blue triangles, *n* = 8). Multifunctionality was analyzed using the FCOM feature of WinList. Significant differences between age groups were denoted by asterisks (*p < 0.05). Black dotted boxes indicate the same multiple cytokine-producing cells in LPMC and PBMC CD8^+^ T subsets.

**Figure 7:**
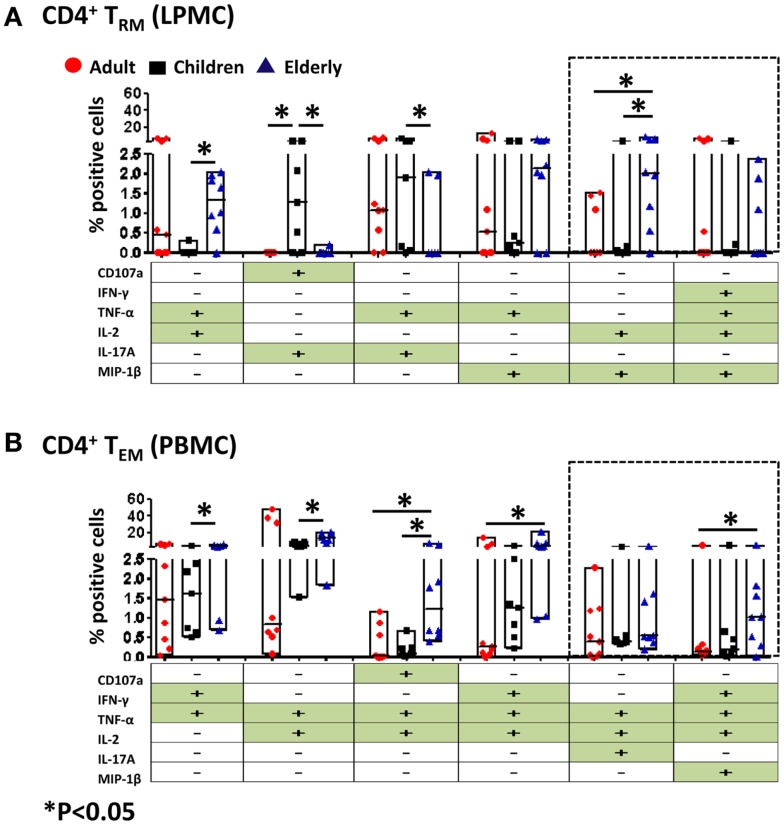
**Multifunctional gastric LPMC CD4^+^ T_RM_ and PBMC CD4^+^ T_EM_ responses to SEB stimulation in adults, children, and the elderly**. Multifunctionality was determined by simultaneous detection of two or more functions performed by CD4^+^ T_RM_ (LPMC) or CD4^+^ T_EM_ (PBMC). Six functions were evaluated: production of five cytokines/chemokines (IFN-γ, TNF-α, IL-2, IL-17A, MIP-1β) and expression of CD107a in response to SEB stimulation. **(A)** Scatter plot showing the six predominant function patterns in LPMC CD4^+^ T_RM_ and **(B)** in PBMC CD4^+^ T_EM_ cells from adults (red circles, *n* = 9), children (black squares, *n* = 7), and the elderly (blue triangles, *n* = 8). Multifunctionality was analyzed using the FCOM feature of WinList. Significant differences between age groups were denoted by asterisks (*p < 0.05). Black dotted boxes indicate the same multiple cytokine-producing cells in LPMC and PBMC CD4^+^ T subsets.

CD4^+^ and CD8^+^ T cells that produce two or more cytokines simultaneously (multifunctional) have enhanced functionality and are more likely to correlate with protection from disease when compared to single cytokine-producing cells (25–28). The induction of multifunctional cells in the human gastric mucosa has not yet been reported and whether these cells play a role in the development or resolution of pathogenesis remains unknown. Thus, we investigated whether CD4^+^ and CD8^+^ T_RM_ (LPMC) obtained from the three age groups had multifunctional properties following SEB stimulation. All possible combinations (64 in total) for five cytokines (IFN-γ, TNF-α, IL-2, IL-17A, MIP-1β) and expression of CD107a were analyzed in multidimensional space using the WinList FCOM function. Similar analyses were performed in CD4^+^ T_EM_ and CD8^+^ T_EM_ (PBMC) populations. The results demonstrated that stimulation elicits multifunctional responses in gastric CD4^+^ T_RM_ and CD8^+^ T_RM_ cells. Similarly, CD4^+^ T_EM_ and CD8^+^ T_EM_ cells demonstrated multifunctionality, which is consistent with previous results from our group as well as others (Figures 6 and 7) (6, 24, 25, 29). For simplicity, shown are only the six highest expressing multifunctional CD8^+^ T_RM_ and CD8^+^ T_EM_ cell groups (Figures 6A,B). Double, triple, quadruple, and quintuple cytokine secreting cells CD8^+^ T_RM_ and CD8^+^ T_EM_ were found in all age groups albeit at different percentages. Interestingly, of the six highest multifunctional groups in gastric CD8^+^ T_RM_ cells, four were also found in CD8^+^ T_EM_ cells (dotted boxes) (Figures 6A,B). We then compared the magnitude of multifunctional T cells between the age groups and identified some differences. A significantly higher percentage of CD8^+^ T_RM_ cells from the elderly contained double (CD107a^+^ MIP-1β^+^) cytokine-producing cells than adults and children (Figure 6A). Similarly, quintuple (CD107a^+^ IFN-γ^+^ TNF-α^+^ IL-2^+^ MIP-1β^+^) cytokine-producing cells in the elderly were significantly more abundant than in adults (Figure 6A). In peripheral blood, differences between the age groups within the multi-cytokine-producing sets were also noted. The percentage of double (TNF-α^+^ IL-2^+^) and triple (IFN-γ^+^ TNF-α^+^ IL-2^+^, as well as CD107a^+^ IFN-γ^+^ MIP-1β^+^) CD8^+^ T_EM_ cells in the elderly group was significantly higher than in children (Figure 6B). Furthermore, the percentage of triple (CD107a^+^ IFN-γ^+^ MIP-1β^+^) CD8^+^ T_EM_ cells was higher in the elderly than in adults (Figure 6B).

We also assessed multifunctionality in CD4^+^ T_RM_ and CD4^+^ T_EM_ cells (Figure 7). Both of these cell populations have the potential to become multifunctional and showed differences in the age groups within various multifunctional sets. Of the six highest multifunctional populations in gastric CD4^+^ T_RM_ cells (LPMC), only two were also found in CD4^+^ T_EM_ cells (PBMC) (dotted boxes) (Figures 7A,B). Double and quadruple cytokine-producing cells were observed in gastric CD4^+^ T_RM_ cells. The percentage of dual producer cells (TNF-α^+^ IL-2^+^ and IL-2^+^ MIP-1β^+^) in the elderly was significantly higher than in children (Figure 7A). Moreover, the percentage of gastric CD4^+^ T_RM_ cells producing IL-2 and MIP-1β in elderly volunteers was also higher than in adults (Figure 7A). Interestingly, the percentage of dual producer CD4^+^ T_RM_ cells consisting of IL-17 (CD107a^+^ IL-17A^+^ and TNF-α^+^ IL-17A^+^) in children was significantly higher than adults and the elderly (Figure 7A). Similarly, PBMC CD4^+^ T_EM_ stimulated with SEB display activated cells that contained double, triple, and quadruple cytokine-producing cells (Figure 7B). In the elderly, a significantly higher percentage of CD4^+^ T_EM_ cells produced double (IFN-γ^+^ TNF-α^+^ or TNF-α^+^ IL-2^+^), triple (CD107a^+^ IL-2^+^ TNF-α^+^ and IFN-γ^+^ TNF-α^+^ IL-2^+^), and quadruple (IFN-γ^+^ TNF-α^+^ IL-2^+^ MIP-1β^+^) cytokines than children and adult volunteers (Figure 7B).

## Discussion

Recent reports have described the presence of T_RM_ cells (CD8^+^) in mucosal surfaces (4–6, 9). These cells, originally described in the mouse model, were very recently identified in the lungs of humans (9), along with CD4^+^ T_RM_ cells, which have been less well characterized. In the stomach mucosa, several immune cells have been described and although different methodologies for isolation of mononuclear cells from biopsies have been reported, the optimal conditions remain largely undefined. In this manuscript, we report an optimized method for the isolation of human gastric leukocytes from stomach biopsies and, using this method, the identification of CD8^+^ T_RM_ and CD4^+^ T_RM_ cells. Moreover, we explored the ability of these cells to produce cytokines following stimulation with various mitogens, as well as demonstrated the multifunctional nature of these responses. Finally, we investigated whether there are differences in the quality and magnitude of the responses in various age groups.

Our cell isolation protocol (LPMC) from gastric biopsies involved the removal of epithelial cells and a mild enzymatic digestion step (13) that was combined with a mild mechanical disruption step using stainless steel beads (BB). This additional step allowed for maximum dislodgement of cells with minimal damage and provided a more consistent and uniform homogenization, decreasing variation between samples and generating higher viable cell yields. Compared to previously published reports, our method, at a minimum doubled the number of viable LPMC isolated from gastric biopsies (12, 30). However, given that biopsies size and weight varies considerably during sampling and that in most studies the biopsy weights have not been reported, the real efficiency of the methods cannot be directly compared. Cell yields expressed as total number of viable cells per milligram of tissue in “dried” biopsies would be optimal to enable this assessment across studies. Interestingly, the number of cells per milligram of tissue in children was higher than in the elderly (Figure 2B). These results were similar to those reported by Bontems et al., who suggested that there was higher cellularity in children than in adults; however, no statistical differences were reported in that study (17). The data from elderly volunteers allowed us to extend the time frame of evaluation and confirmed that as the age of the volunteers increased, the number of mononuclear cells isolated in the stomach decreased.

Consistent with previous reports, the frequency of CD3^+^ cells was lower as a percentage of total LPMC cells in the gastric mucosa than in PBMC (19, 31). Additionally, CD4^+^ and CD8^+^ T cells from gastric LPMC were found at similar frequencies as reported by others (14, 16) and the vast majority of gastric CD8^+^ and CD4^+^ T cells showed a T_EM_ phenotype (CD62L^−^, CD45RA^−^). These results provide evidence that the optimized cell isolation method described in the present manuscript did not result in cell subset selection bias. Therefore, these cells appeared to be of the newly defined tissue-T_RM_ cells in human intestinal tissues (32). This presumption was confirmed by investigating the expression of hallmark receptors for these cells, including CD103 and CD69, which were expressed by T cells isolated from gastric tissues. Interestingly, differences were noted between CD8^+^ T_RM_ and CD4^+^ T_RM_ cells in gastric tissues. For example, the large majority of CD8^+^ T_RM_ cells co-expressed CD103 and CD69, whilst only a small proportion expressed CD103 alone. In contrast, only ~35% of CD4^+^ T_RM_ cells co-expressed CD103 and CD69. Therefore, CD8^+^ T_RM_ and CD4^+^ T_RM_ in the human gastric lamina propria exhibited a differential expression pattern of molecules reported to define T_RM_ in other human mucosal tissues (7, 8). These observations suggest that CD4^+^ T_RM_ cells are a more heterogeneous and complex population than CD8^+^ T_RM_, possibly composed by various subsets. Future studies are necessary to address this important question. Of note, CD4^+^ and CD8^+^ T_RM_ were present in children, adult, and the elderly at similar frequencies.

Migration of immune cells from peripheral blood to gut tissues is driven by the expression of tissue-specific homing receptors such as integrin α4β7 and CCR9 (33, 34). Since gastric T_RM_ cells are expected to permanently reside in this tissue, we reasoned that up-regulation of CD103, which binds to E-cadherin and allows homing at the mucosal level, will result in down-regulation of integrin α4β7. Consistent with this, CD8^+^ T_RM_ and CD4^+^ T_RM_ cells showed a significant down-regulation of integrin α4β7 compared to its expression levels in CD8^+^ T_EM_ and CD4^+^ T_EM_ (PBMC). These results are consistent with data reported in the mouse model in tissues isolated from the small intestine (32, 35). Similar results were seen in adults, children, and the elderly. Whether CCR9 is also down-regulated in CD8^+^ and CD4^+^ T_RM_ cells remains to be explored. The fact that we were able to identify CD8^+^ T_RM_ and CD4^+^ T_RM_ cells in LPMC, which contain cells from the lamina propria, suggests that T_RM_ cells are either constantly mobilizing between the epithelial and lamina propria layers of the stomach or reside mainly in the lamina propria layer. In an attempt to address this question, we assessed the IEL fraction in some volunteers and identified mainly CD8^+^ T cells, most of which co-expressed CD103 and CD69 (data not shown). CD4^+^ T cells were also identified in the IEL fraction, but at such low frequencies that we were unable to ascertain their levels of expression of CD103 and CD69 (data not shown). It is reasonable to speculate that T_RM_ cells mainly reside in the lamina propria and once they migrate to the epithelium (i.e., becoming part of the traditional IEL subset) are unable to re-enter the lamina propria. In the latter scenario, T_RM_ cells in the lamina propria will constantly supply cells that migrate to the epithelial layer. This would require a change in expression of homing markers and would suggest that, in addition to CD103, other receptor(s) yet to be identified is(are) involved in the homing of these cells from the lamina propria to the epithelium. At this time our data are unable to determine which of these hypotheses is correct. However, it is likely that T_RM_ cells shuttle between these two compartments working as sentinel cells and when a specific antigen is encountered, these cells are rapidly activated, producing cytokines and acquiring CTL activity.

CD8^+^ T_RM_ cells have been described in mice as well as humans, and reactivity of these cells to antigens derived from pathogens has been demonstrated (4–6, 9). We identified CD8^+^ T_RM_ and CD4^+^ T_RM_ cell in the stomach of volunteers confirmed to be *H. pylori* negative (as determined by CLO test) (36). While *H. pylori* is well recognized for its role in the development of gastritis, peptic ulcer, and adenocarcinoma, it does not affect the composition of the gastric community (37). The gastric microbiota has been shown to contain a diverse community of 128 phylotypes (37), which could provide the underlying T cells with antigen(s) to regulate their development. It can be speculated that unidentified infectious agents or the gastric microbiota, through conserved epitopes that resemble those of pathogens, play a role in the development of T_RM_ cells (38). Whichever the event(s) that triggers their development, it appears that they occur at a young age, since even the youngest children evaluated in our studies (i.e., 7-year-old) showed the presence of these unique cells. Future experiments designed to address these questions will include the investigation of the role of the microbiota in the development of T_RM_ cells and a comparison of the cytokine production by CD4^+^ T_RM_ and CD8^+^ T_RM_ cells from *H. pylori* positive and healthy volunteers following stimulation with *H. pylori* antigens.

Gastric CD8^+^ T_RM_ and CD4^+^ T_RM_ cells obtained from biopsies of children, adults, and the elderly were responsive to SEB and anti-CD3/CD28 beads stimulations by secreting Th1 cytokines (IL-2, IFN-γ, TNF-α, IL-17A, MIP-1β) and up-regulating the cytotoxicity marker CD107a. These results confirm and extend studies in which gastric CD4^+^ T cells obtained from healthy adults (*H. pylori* negative) secreted Th1 cytokines (IFN-γ and TNF-α) when stimulated with PMA/Ionomycin (16, 31). Interestingly, CD8^+^ T_RM_ cells appeared to produce cytokines constitutively; a higher percentage of T_RM_ cells cultured in media only showed cytokine production compared to CD8^+^ T_EM_ cells. Similar results, albeit not statistically significant, were identified in CD4^+^ T_RM_ cells. These observations suggest that T_RM_ cells are more prone to activation and possibly have a lower antigenic threshold for stimulation than their peripheral blood counterparts. However, it is important to consider that while the volunteers were *H. pylori* negative, they were referred for EGD due to the presence of clinical symptoms (e.g., dysphagia, heartburn, GERD, etc.). Therefore, to determine if gastric T_RM_ cells were activated in response to an inflammatory environment resulting from the underlying clinical condition(s), we stratified the baseline cytokine levels based on the pathology findings from each volunteer (normal and “mild inflammation”) (Figure S2 in Supplementary Material). Neither CD8^+^ T_RM_ nor CD4^+^ T_RM_ cells showed statistically significant differences between the normal and mild inflammation groups. These results support the idea that T_RM_ cells show a persistent activation state in “normal volunteers.” An alternative explanation for the persistent activation state of T_RM_ cells could involve the role of the gastric microbiota. Thus, future studies should be directed to explore this and other alternative explanations. Whether the higher percentage of cells producing cytokines and up-regulating CD107a spontaneously have a deleterious effect at the gastric mucosal level or that this enhanced inflammatory environment benefits the host by limiting colonization with pathogens remains to be explored. Overall, CD8^+^ T_RM_ cells from adult, children, and the elderly responded to the stimuli and the cytokine production was higher compared to PBMC, but more evident in adults and elderly than in children.

There is little information on the induction of local immune responses in the gastric mucosa from children (39, 40). Few studies have evaluated the cytokine responses in the gastric mucosa of this age group and the results are contradictory (17, 41, 42). One study found that lower levels of IFN-γ were produced in culture supernatants of gastric mucosa tissues from children compared to adults, but no differences in TNF-α, IL-2, or IL-10 were detected regardless of their *H. pylori* status (17). On the other hand, a recent study showed that in *H. pylori* infected children, the gastric concentration of IL-1α and TNF-α were significantly higher than that in infected adults whereas IL-2, IL-12p70, and IFN-γ were lower in infected children than in infected adults (42). Of note, differences in cytokine profiles were observed between infected and uninfected individuals in both age groups (42). Epidemiological studies have also suggested that unlike adults, children rarely develop peptic ulcers or gastric atrophy (43–45). This suggests that children may display a unique immunological milieu that limits gastric mucosal damage. In our study, even though the phenotype and abundance of gastric CD8^+^ T_RM_ and CD4^+^ T_RM_ in children were similar to those of adults and the elderly, their responses were different. CD4^+^ T_RM_ and CD8^+^ T_RM_ cells from children responded only moderately to the mitogenic stimulations and secreted lower amounts of cytokines than their adult and the elderly counterparts. Our results are consistent, and markedly extend, previous studies demonstrated that gastric T cells from children are less responsive to stimulation than adults. Moreover, our results provide novel information on cells isolated from elderly subjects. Additionally, it has been shown that Th1 and Th17 responses in children are down-regulated, resulting in reduced gastritis due to *H. pylori* infections (46). This observation contrasts with that of adults, in whom *H. pylori* infections usually result in significant inflammation. Furthermore, in children who are positive for *H. pylori*, the levels of regulatory T cells (T_regs_) and IL-10 secreting cells in the gastric mucosa are higher than in *H. pylori* infected adults (46, 47). Therefore, these observations suggest that in children the regulatory mechanisms at the gastric level are more active than in adults and this could contribute to the limited reactivity identified in CD4^+^ and CD8^+^ T_RM_ cells. Future studies involving the investigation of the presence and functional properties of T_regs_ in the gastric mucosa of children as compared to adults/elderly will shed light into this important question.

Multifunctionality analysis following SEB stimulation confirmed that T_RM_ are multifunctional and also reinforced the idea that age is a significant factor. Interestingly, various multi-cytokine production patterns demonstrated that in the elderly a higher percentage of cells produced multiple cytokines than in children, suggesting that elderly cells are more reactive to stimulation. This data further confirmed and extend the observations in this manuscript that cells from children are less susceptible to activation. Of note, there were a few instances in which cells from children produced more cytokines than those isolated from the elderly (e.g., CD4^+^ T_RM_ dual producers CD107a and IL-17A, as well as TNF-α and IL-17A). This reinforces the indication that cytokine production is age-related.

In summary, we developed a consistent method for isolation of immune cells from the gastric biopsies that increased cell yields and allowed the identification of CD8^+^ T_RM_ and CD4^+^ T_RM_ cells in children, adults, and the elderly. We demonstrated that these cells were functional and responsive to various categories of stimulants. Finally, we show that gastric cells of children respond differently to stimuli than adults and the elderly in terms of cytokines and multi-cytokine production suggesting that unique regulatory mechanisms are operative in the children’s gastric mucosa.

## Conflict of Interest Statement

The authors declare that the research was conducted in the absence of any commercial or financial relationships that could be construed as a potential conflict of interest.

## Supplementary Material

The Supplementary Material for this article can be found online at http://journal.frontiersin.org/Journal/10.3389/fimmu.2014.00294/abstract
