# Supplementary material for: Characterization and Functional Properties of Gastric Tissue-Resident Memory T Cells from Children, Adults, and the Elderly
Source: Front Immunol. 2014 Jun 19;5:294. doi: 10.3389/fimmu.2014.00294 (PMC4062881; doi:10.3389/fimmu.2014.00294)
Supplement: Supplementary file 1 [file Presentation1.PPTX]

## Slide 1
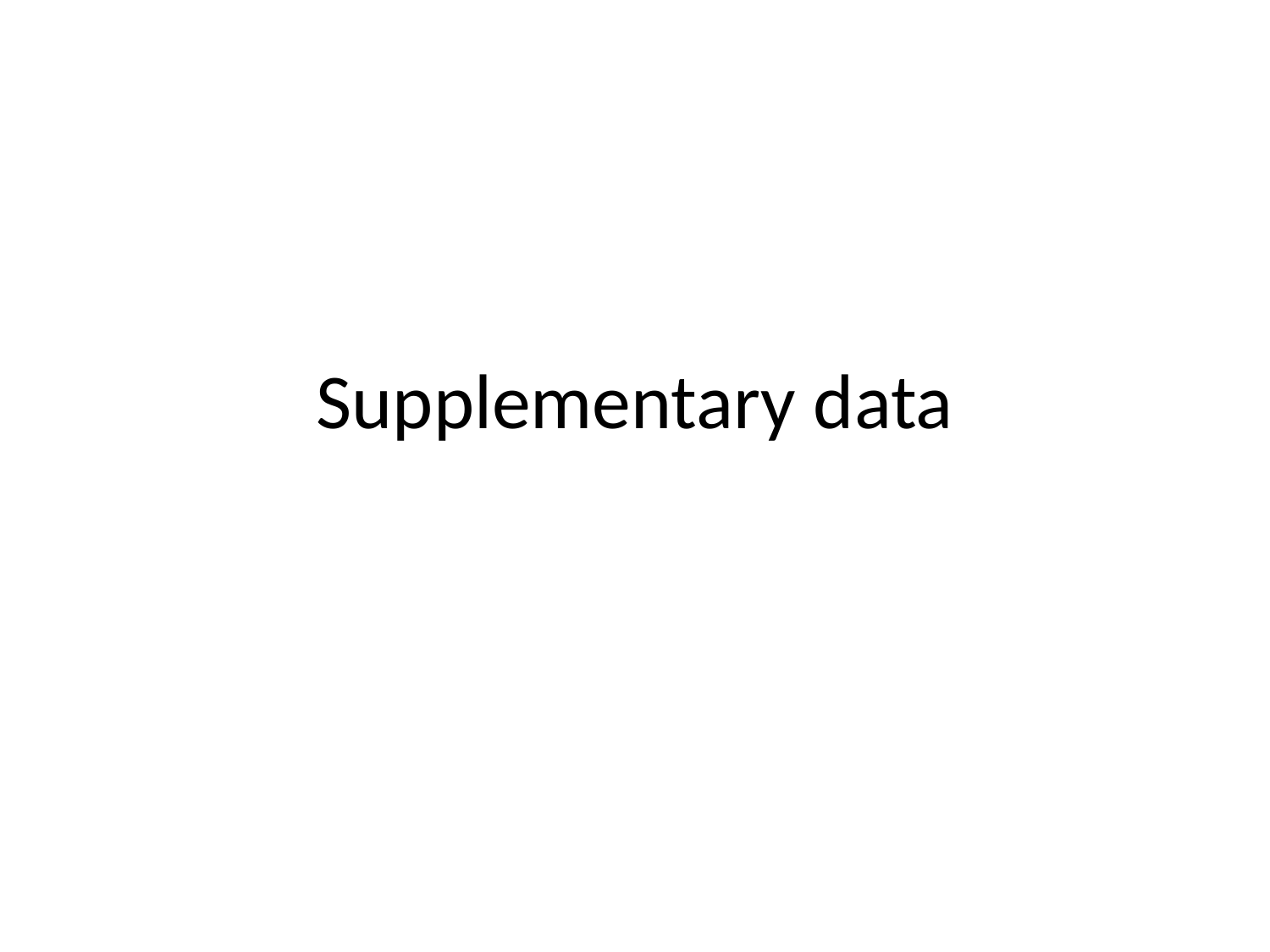

# Supplementary data

## Slide 2
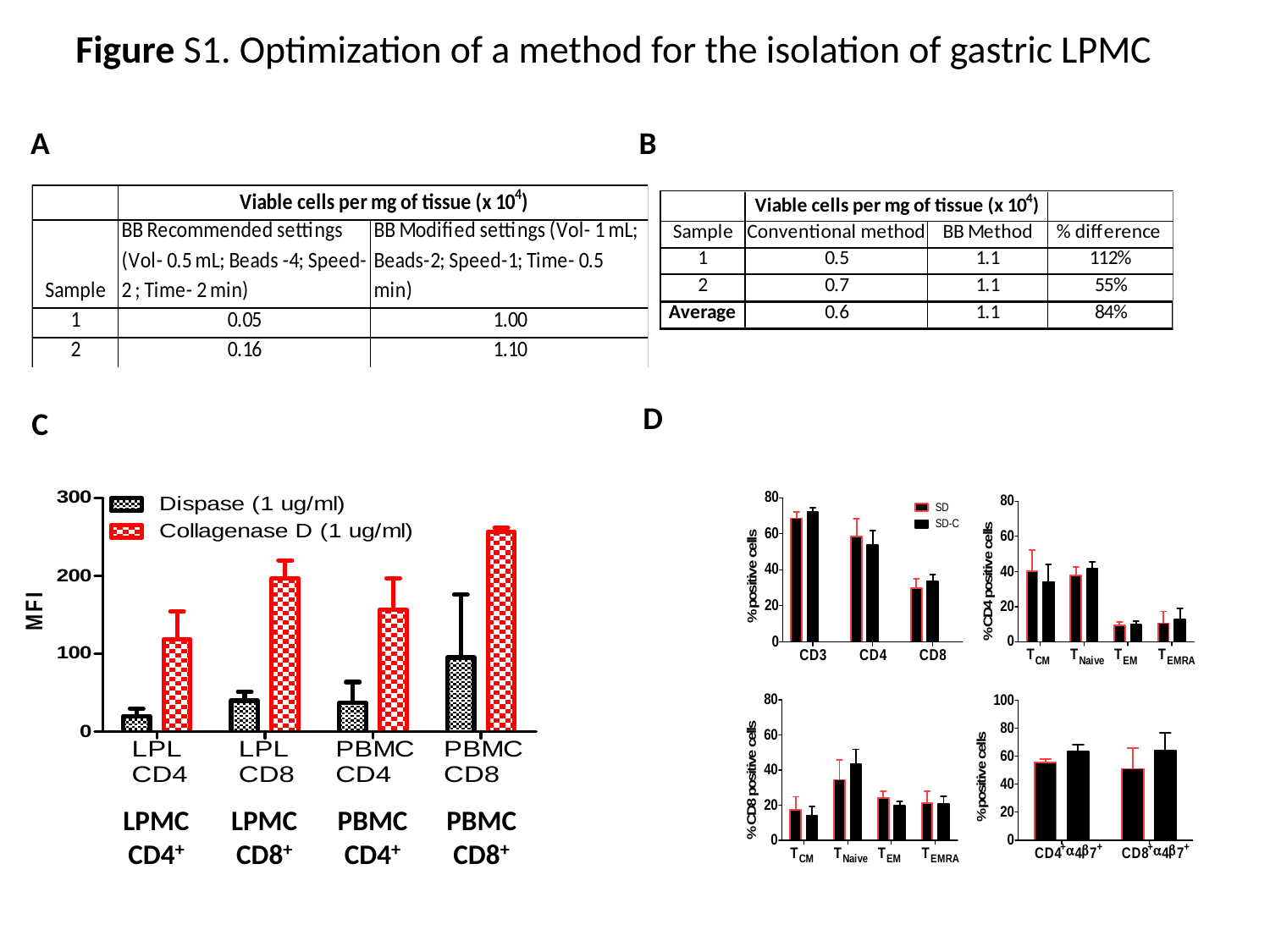

# Figure S1. Optimization of a method for the isolation of gastric LPMC
B
A
D
C

## Slide 3
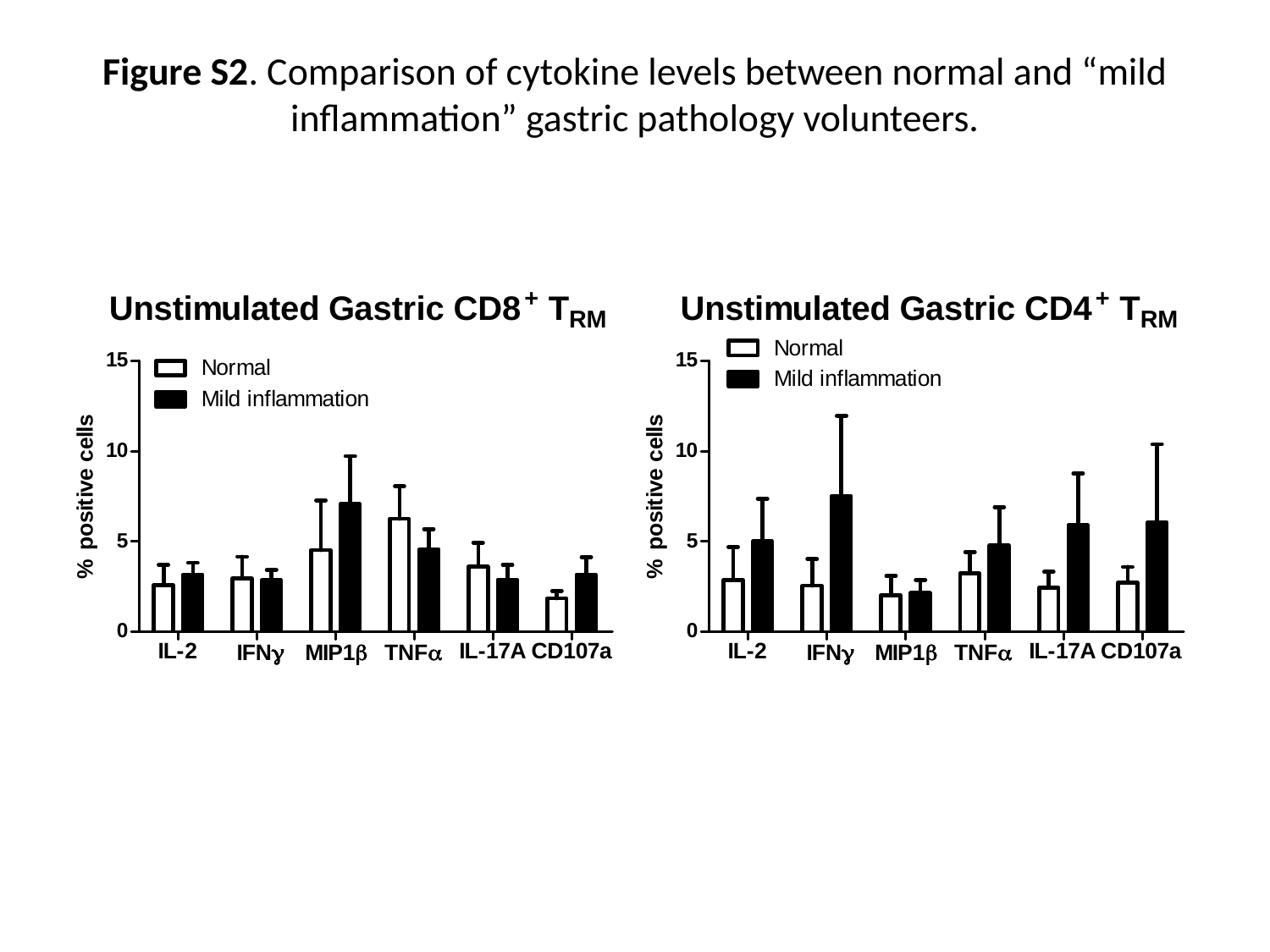

# Figure S2. Comparison of cytokine levels between normal and “mild inflammation” gastric pathology volunteers.

## Slide 4
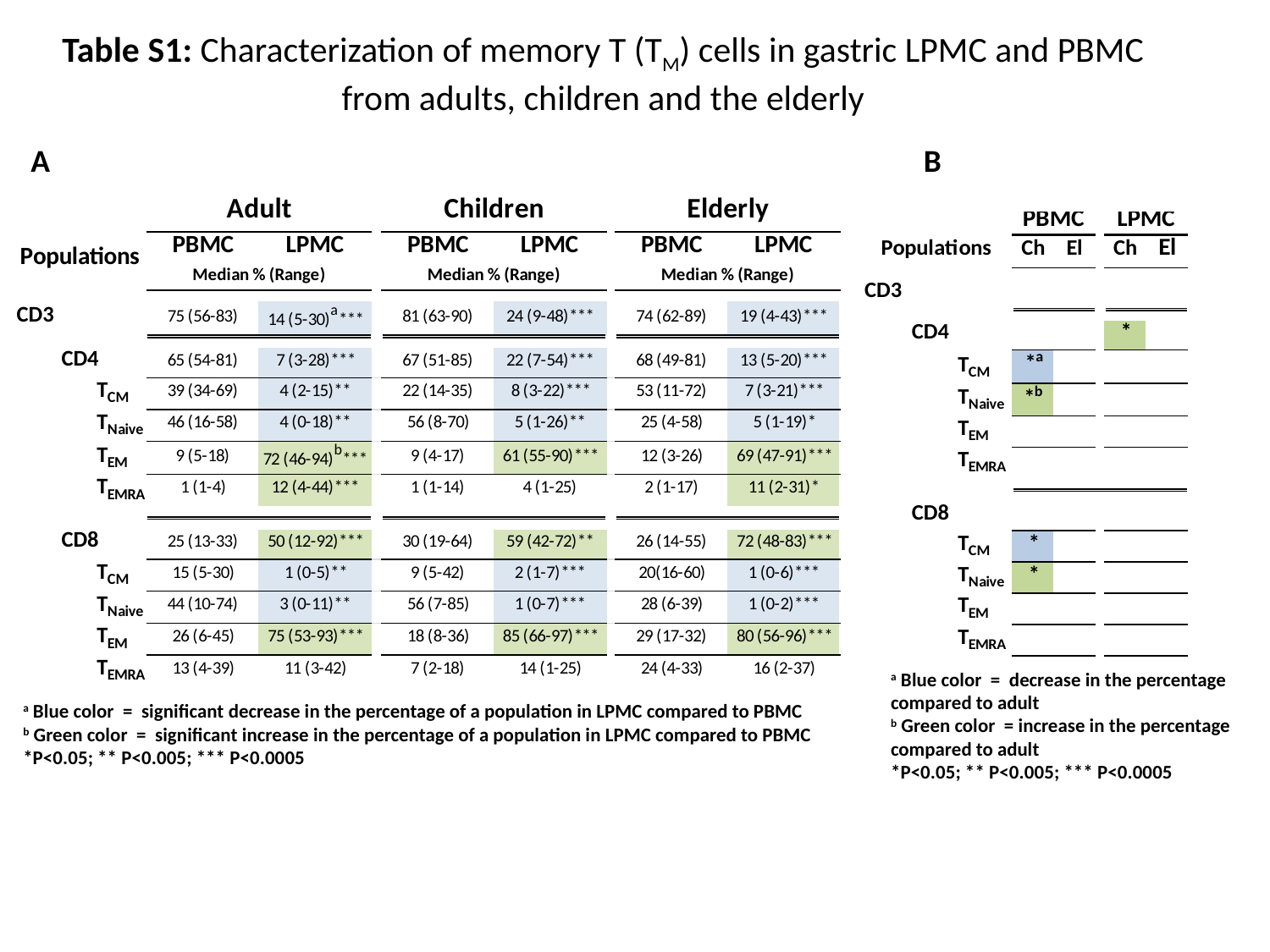

Table S1: Characterization of memory T (TM) cells in gastric LPMC and PBMC from adults, children and the elderly
A
B
a Blue color = significant decrease in the percentage of a population in LPMC compared to PBMC
b Green color = significant increase in the percentage of a population in LPMC compared to PBMC
*P<0.05; ** P<0.005; *** P<0.0005
a Blue color = decrease in the percentage compared to adult
b Green color = increase in the percentage compared to adult
*P<0.05; ** P<0.005; *** P<0.0005
